# Validation and Psychometric Evaluation of the Persian Version of the Nurse Intuition Patient Deterioration Scale: A Methodological Study

**DOI:** 10.1002/nop2.70616

**Published:** 2026-05-25

**Authors:** Reza Nemati‐Vakilabad, Mohammad Javad Jafari, Alireza Mirzaei, Filip Haegdorens, Nargess Ramazanzadeh, Sevda Gardashkhani, Mehraban Shahmari

**Affiliations:** ^1^ Students Research Committee, School of Nursing and Midwifery Ardabil University of Medical Sciences Ardabil Iran; ^2^ Department of Emergency Nursing, School of Nursing and Midwifery Ardabil University of Medical Sciences Ardabil Iran; ^3^ Student Research Committee, School of Nursing and Midwifery Tabriz University of Medical Sciences Tabriz Iran; ^4^ Workforce Management and Outcome Research in Care (WORC) Group; Centre for Research and Innovation in Care (CRIC) Wilrijk Belgium; ^5^ Department of Medical‐Surgical Nursing, School of Nursing and Midwifery Ardabil University of Medical Sciences Ardabil Iran; ^6^ Department of Medical‐Surgical Nursing, Faculty of Nursing and Midwifery Tabriz University of Medical Sciences Tabriz Iran

**Keywords:** deterioration, hospital, intuition, nurse, psychometric, scale

## Abstract

**Aim:**

Early recognition and intervention in cases of clinical deterioration are essential to preventing significant harm to patients, with nurses playing a crucial role in this process. The Nurse Intuition Patient Deterioration Scale (NIPDS) was developed to assess various aspects of a patient's condition, enabling nurses to identify potential issues even when vital signs do not indicate an urgent concern. This study aims to validate the Persian version of the NIPDS (NIPDS‐P) and assess its psychometric properties based on the assessments of 250 hospitalized patients whose clinical status was evaluated by clinical nurses in Iran.

**Design:**

A two‐phase validation process involved the NIPDS‐P ratings completed by clinical nurses for 250 hospitalized patients from multiple departments, including general wards, intensive care units, and emergency departments. The nurses completed the NIPDS‐P for each patient based on their intuitive clinical assessment.

**Methods:**

The study assessed the scale's reliability, construct validity, and face validity through quantitative and qualitative methods following COSMIN guidelines.

**Results:**

The content validity was confirmed by experts, as indicated by a Content Validity Index (CVI) of 0.92 and a Content Validity Ratio (CVR) exceeding 0.99. The NIPDS‐P exhibited high internal consistency, with both Cronbach's Alpha and McDonald's Omega values exceeding 0.95. Exploratory factor analysis (EFA) was conducted using the common‐factor method with maximum likelihood extraction, which supported a unidimensional structure (one factor, eigenvalue = 6.567, explaining 72.97% of the variance). Confirmatory factor analysis (CFA), employing the diagonally weighted least squares (DWLS) estimator with polychoric correlations, confirmed this structure, yielding acceptable fit indices: CFI = 0.980, TLI = 0.975, RMSEA = 0.078 (90% CI [0.054, 0.102]), and SRMR = 0.041.

**Conclusion:**

The NIPDS‐P is reliable and valid for evaluating nurse intuition regarding patient deterioration in Iran. While its strengths highlight its potential to enhance nursing practice, further research is needed to explore its applicability in broader clinical settings and diverse patient populations. The high alpha (0.96) may indicate some item redundancy, and the cut‐off score of ≥ 5 should be considered provisional pending ROC analysis against objective clinical outcomes.

## 1 Introduction

Early recognition and response to clinical deterioration are critical in preventing severe patient harm, with nurses playing a vital role in this process (Liu et al. 2024). In clinical decision‐making, nurses primarily rely on objective patient vital signs such as heart rate and blood pressure rather than relying solely on intuition (Ignatavicius and Andersen 2024). To systematically assess a patient's condition, Haegdorens et al. (2023, 2024) in Belgium developed the Nurse Intuition Patient Deterioration Scale (NIPDS). This tool evaluates nine aspects of a patient's condition, including the ability to express oneself, facial expressions, and alertness. Nurses score each item from 0 (absent) to 2 (strongly present), yielding a total score between 0 and 18. A score of 5 or higher categorizes a patient as high‐risk, necessitating immediate attention and intervention.

The NIPDS is considered a promising tool for improving patient outcomes by helping nurses detect potential issues even when standard vital signs appear normal. Some research suggests it may be more effective than established tools like the National Early Warning Score (NEWS), which, despite its widespread use, can sometimes miss subtle clinical changes (Haegdorens et al. 2024). For instance, a patient experiencing an acute myocardial infarction might have a low NEWS score, potentially delaying concern. Beyond NEWS, other scores, such as the Modified Early Warning Score (MEWS), are also used in critical care. These scales are typically grounded in objective physiological data rather than subjective clinical judgement (Marshall 2020).

However, the effective implementation of a clinically intuitive scale, such as the NIPDS, requires rigorous validation. The accuracy of such tools can be influenced by nursing experience, and scores may vary in settings with less experienced staff, potentially leading to inaccurate assessments (Haegdorens et al. 2024). Therefore, ensuring the validity and reliability of the scale through psychometric evaluation is crucial for its successful application in patient care (Williams 2022).

This study aims to translate the Nurse Intuition Patient Deterioration Scale (NIPDS) into Persian and conduct a thorough psychometric evaluation of the translated version. The translation process will consist of both forward and backward translations by bilingual experts, followed by a review and consensus process to ensure linguistic and cultural equivalence. The validation process will assess the scale's reliability, validity and sensitivity to change among a sample of Iranian healthcare professionals and patients. Specific local healthcare challenges, such as high nurse‐to‐patient ratios, substantial staff workloads, and frequent nursing personnel shortages, highlight the need for this research in the Iranian context. These factors can hinder the continuous monitoring of subtle clinical changes, increasing the risk of overlooking early signs of patient deterioration.

Additionally, the unique demographics and disease patterns present within the Iranian patient population emphasize the importance of validating clinical tools tailored to this specific setting. This research aims to provide a standardized and reliable instrument to support clinical judgement under these constrained conditions. Ultimately, by equipping nurses with a validated framework for identifying clinical decline, this study seeks to enhance the quality of healthcare delivery and improve patient outcomes in Iran.

## 2 Methods

### 2.1 Study Design and Setting

This methodological study utilized a two‐phase design to validate the Nurse Intuition Patient Deterioration Scale (NIPDS) within the context of nursing practice in Iran. The design and reporting followed the COSMIN (Consensus‐based Standards for the selection of health Measurement Instruments) checklist for patient‐reported outcome measurement instruments. The research was conducted across various clinical settings, including general wards, intensive care units (ICUs), and emergency departments at several hospitals in Ardabil, Iran. These settings were selected to ensure a comprehensive representation of clinical environments and to accurately reflect the real‐world scenarios in which nurses assess patient deterioration.

### 2.2 Participants

The study was based on NIPDS‐P ratings completed by clinical nurses for 250 hospitalized patients admitted to the participating hospitals during the study period. The nurses completed the NIPDS‐P for each patient based on their intuitive clinical judgement. Inclusion criteria for patients whose clinical status was assessed were as follows: (a) age 18 years or older; (b) ability to communicate effectively and provide written informed consent for the use of their health data in the study; and (c) admission to a medical or surgical unit, thereby enabling nursing staff to conduct a comprehensive clinical evaluation of their condition. Exclusion criteria included: (a) severe cognitive impairments (e.g., dementia or significant neurological disorders) that precluded the provision of informed consent; (b) receipt of palliative or end‐of‐life care; and (c) inability to provide informed consent due to language barriers, non‐verbal status, or other factors. For the nursing collaborators: A total of 50 clinical nurses from the same departments voluntarily participated in completing the NIPDS‐P for the patients. Nurses were included if they had at least 6 months of clinical experience in their respective units.

### 2.3 Data Collection and Measures

The demographic characteristics form included data on the patients' age, gender, marital status, reason for admission and clinical unit as documented in hospital records.

### 2.4 Nurse Intuition in Patient Deterioration Scale

The Nurse Intuition in Patient Deterioration Scale (NIPDS), developed by Haegdorens et al. (Haegdorens et al. 2023, 2024), comprises nine items to assess nurses' intuitive perceptions of patient deterioration. Respondents assessed each item on a 3‐point Likert scale: a score of 0 indicated the absence of the indicator, a score of 1 suggested a moderate presence, and a score of 2 denoted a strong presence. The cumulative score from these items provided valuable insights into nurses' intuitive abilities. A score of 5 or higher was adopted from the original NIPDS study (Haegdorens et al. 2023) as the threshold indicating significant nurse intuition concerning potential patient deterioration. It is important to note that this cut‐off score was not empirically validated in the present study, as we did not collect objective clinical outcome data required for ROC analysis (see rationale below). Therefore, the cut‐off of ≥ 5 should be considered provisional for the Persian version pending future criterion validity testing. According to Haegdorens et al., this assessment tool demonstrated robust psychometric properties, including high reliability, as evidenced by a Cronbach's alpha exceeding 0.80, indicating internal consistency. Its validity was supported by factor analysis and correlations with established measures, confirming its effectiveness in capturing the intended psychological constructs. Furthermore, the tool showed solid test–retest reliability, with a correlation coefficient exceeding 0.75, ensuring consistent scores over time.

### 2.5 Translation and Cross‐Cultural Adaptation

The translation and cross‐cultural adaptation of the NIPDS were conducted in accordance with the International Test Commission (ITC) guidelines for translating and adapting tests. The process comprised six methodical steps:

1. *Initial forward translation*: Two independent bilingual translators, both native Farsi speakers with proficiency in English, translated the original English version of the NIPDS into Farsi. One translator, a healthcare professional (specifically a nurse), was familiar with the relevant constructs, while the other was a professional translator without prior exposure to the scale's objectives. This stage produced two forward‐translated versions (T1 and T2).
2. *Synthesis of forward translations*: The two translators, along with the research team, convened to review T1 and T2. They engaged in discussions to address and resolve any discrepancies in terminology, conceptual meaning, and cultural significance, ultimately merging the two versions into a single reconciled Farsi translation (T‐12).
3. *Back‐translation*: The synthesized Farsi version (T‐12) was subsequently back‐translated into English by two additional independent bilingual translators, both native English speakers fluent in Farsi and unaware of the original instrument. This process yielded two back‐translated versions (BT1 and BT2).
4. *Expert committee review*: An expert committee was formed, comprising the forward and back translators, a methodologist, and a language professional. They systematically compared the original NIPDS with the back‐translated versions (BT1 and BT2) as well as the reconciled Farsi version (T‐12). The committee critically assessed all reports, addressing any semantic, idiomatic, experiential, or conceptual inconsistencies and reached a consensus on a pre‐final version of the Persian NIPDS.
5. *Review by the original developer*: The pre‐final Persian version was submitted to Professor Haegdorens, the original developer of the NIPDS, for review and validation. The developer confirmed that the translated version maintained the original instrument's intent, conceptual integrity, and item structure.
6. *Pretesting and cognitive debriefing*: The pre‐final version was administered to a panel of 15 practicing nurses' representatives of the target population. Participants were asked to complete the scale and provide feedback regarding the clarity, comprehensibility, and cultural appropriateness of each item and the instructions. Based on their feedback, minor adjustments were made to the wording to finalize the Persian NIPDS. A completed ITC translation/adaptation checklist is provided as File S2.

### 2.6 Psychometric Testing (Structured According to COSMIN Headings)

#### 2.6.1 COSMIN Box I: Face Validity (Relevance and Comprehensibility)

A focus group method was employed to assess face validity, involving ten practicing nurses from diverse clinical backgrounds. The focus group sessions were structured to facilitate open discussion and gather qualitative feedback on the items of the assessment scale. Each participant was presented with the scale and asked to evaluate the clarity, relevance and appropriateness of the language used in the items. Participants were encouraged to share their personal experiences and perceptions regarding how well the items aligned with their clinical practice. This approach not only fostered a collaborative environment for feedback but also provided insights into the effectiveness of the items in addressing the needs and realities of nursing practice. The qualitative data collected during these sessions were systematically analysed to identify common themes, reinforcing the scale's face validity as perceived by its target users.

#### 2.6.2 COSMIN Box II: Content Validity

The content validity of the NIPDS‐P was evaluated using the Content Validity Index (CVI) and the Content Validity Ratio (CVR). Following the COSMIN‐recommended method, ten experts assessed each item based on its relevance and necessity. To establish the validity of individual items, a cut‐off threshold of 0.78 or higher was set for the CVI; items meeting or exceeding this criterion were considered valid. The importance of each item was assessed via the CVR, where a threshold of 0.62 or higher indicated that an item was deemed essential (Shi et al. 2012).

#### 2.6.3 COSMIN Box II: Content Validity (Relevance, Comprehensiveness and Necessity)

##### 2.6.3.1 Item Analysis

Prior to conducting the EFA, an item analysis was performed on the first subsample (*n* = 150 patient ratings) to evaluate the properties of each item and its relationship to the total score. To evaluate the distributional properties of the data, floor and ceiling effects were examined for both individual items and the total scale score. The ceiling effect was defined as the percentage of respondents who achieved the highest possible score on an item (score of 2) or on the total scale (score of 18). Following recommendations in the psychometric literature, floor and ceiling effects below 20% were considered acceptable, although higher thresholds have also been proposed for instruments with a restricted response range (McHorney and Tarlov 1995). Multivariate outliers were assessed using Mahalanobis distance, with a critical value of *χ* ^2^ (9) = 27.88 (*p* > 0.001). No case exceeded this threshold, indicating the absence of multivariate outliers. Additionally, the dataset contained no missing responses, as all nine items of the NIPDS‐P were completed in their entirety by all participants. The results indicated that all items had good variability and correlated strongly with the total score, with item‐total correlations ranging from 0.75 to 0.85. No items were identified that would negatively impact the scale's reliability; therefore, all nine items were retained for the factor analysis.

#### 2.6.4 COSMIN Box III: Construct Validity—Structural Validity

To assess the construct validity of the NIPDS‐P, we employed a two‐step approach involving Exploratory Factor Analysis (EFA) followed by Confirmatory Factor Analysis (CFA). EFA was conducted using the common‐factor model with Maximum Likelihood (ML) extraction, as recommended by COSMIN for ordinal data with more than five response categories. Principal Component Analysis (PCA) was not used.

Before the analysis, the suitability of the data was evaluated using the Kaiser‐Meyer‐Olkin (KMO) measure and Bartlett's test of sphericity. The KMO value was 0.951, and Bartlett's test was significant (*χ* ^2^ = 1965.017, df = 36, *p* < 0.001), confirming the appropriateness of the data for factor analysis. Maximum Likelihood Estimation (MLE) was employed to extract factors. Parallel analysis (based on 1000 random datasets) was conducted to determine the number of factors to retain. The parallel analysis indicated that only the first eigenvalue (6.567) exceeded the 95th percentile random eigenvalue (1.32), supporting a one‐factor solution. Communalities and factor loadings were examined to determine the strength of item correlations with extracted factors. A minimum factor loading of 0.4 and communalities above 0.4 were considered indicative of strong relationships. All nine items were retained based on factor loadings > 0.84, communalities > 0.70 and theoretical alignment with the original scale.

##### 2.6.4.1 Confirmatory Factor Analysis (CFA)—Ordinal Data Method

Following the EFA, CFA was carried out to test the factor structure identified previously. Given the ordinal nature of the items (3‐point Likert scale), the assumption of multivariate normality was violated, as confirmed by examining the response distributions and calculating Mardia's multivariate skewness and kurtosis. Therefore, the analysis was conducted using Diagonally Weighted Least Squares (DWLS) estimation with polychoric correlations, which is specifically recommended for ordinal data because DWLS does not assume normality and instead uses the asymptotic covariance matrix derived from polychoric correlations (Li 2016; Rhemtulla et al. 2012). This approach is more appropriate for Likert‐type scales with fewer than five response categories, as Maximum Likelihood (ML) estimation would produce biased standard errors and inflated chi‐square statistics under non‐normal ordinal conditions. Several goodness‐of‐fit indices were calculated to assess model fit, including the chi‐square statistic, chi‐square to degrees of freedom ratio (*χ* ^2^/df), Standardized Root Mean Square Residual (SRMR), Root Mean Square Error of Approximation (RMSEA) with its 90% confidence interval, Goodness of Fit Index (GFI), Normed Fit Index (NFI), Tucker‐Lewis Index (TLI), Incremental Fit Index (IFI), Relative Fit Index (RFI), Comparative Fit Index (CFI), Adjusted Goodness of Fit Index (AGFI) and Parsimony Normed Fit Index (PNFI). Cut‐off values for model fit indices were selected based on established recommendations in the psychometric literature. Specifically, the following criteria were adopted: Root Mean Square Error of Approximation (RMSEA) < 0.08 was considered acceptable and < 0.05 good; Comparative Fit Index (CFI) and Tucker‐Lewis Index (TLI) > 0.90 were considered acceptable and > 0.95 good; Standardized Root Mean Square Residual (SRMR) < 0.08 was considered acceptable (Schermelleh‐Engel et al. 2003). These thresholds were chosen because they are widely recognized in structural equation modelling for ordinal data. Additionally, while Hu and Bentler (1999) proposed more stringent cut‐offs (e.g., CFI > 0.95, RMSEA < 0.06), these were originally developed for continuous data under normality assumptions. Given the ordinal nature of our data (3‐point Likert scale) and the use of DWLS estimation, the more lenient thresholds proposed by Schermelleh‐Engel et al. (2003) are more appropriate to avoid over‐rejecting well‐fitting models (Marsh et al. 2004). A non‐significant chi‐square result indicated a well‐fitting model, RMSEA values below 0.08, and supportive fit indices (GFI, NFI, IFI, RFI, CFI) exceeding 0.90. Additionally, the AGFI and PNFI values are above 0.80 and 0.50, respectively. The significance of factor loadings was assessed to confirm the strength of relationships between items and the extracted factor, with a threshold of *p* < 0.001 indicating statistical significance.

#### 2.6.5 COSMIN Box IV: Construct Validity—Convergent Validity

To assess the convergent validity of the NIPDS‐P, we employed the Fornell and Larcker criterion. We hypothesized that the NIPDS‐P would show moderate to high convergent validity (CR > 0.70, AVE > 0.50). Following the CFA, we calculated the Composite Reliability (CR) and Average Variance Extracted (AVE). The CR was 0.958, and the AVE was 0.579, both exceeding the hypothesized thresholds, supporting convergent validity. Effect sizes for factor loadings ranged from 0.78 to 0.85 (large effects).

#### 2.6.6 COSMIN Box V: Reliability (Internal Consistency, Test–Retest and Measurement Error)

##### 2.6.6.1 Internal Consistency

The internal consistency of the NIPDS‐P was evaluated based on the 250 patient ratings completed by nurses. The responses were analysed using Cronbach's Alpha and McDonald's Omega. In addition, mean inter‐item correlations were calculated to assess for potential item redundancy given the high alpha (0.956). The mean inter‐item correlation was 0.68 (range 0.62–0.74), suggesting a homogenous scale with some possible overlap among items. A threshold of 0.70 was established to ensure acceptable reliability.

##### 2.6.6.2 Test–Retest Reliability and Measurement Error

To assess the test–retest reliability, a subset of 40 patients was re‐evaluated by the same nurses 2 weeks after the initial administration. The interval was chosen to ensure that patients' clinical status remained relatively stable while minimizing memory effects. The Intraclass Correlation Coefficient (ICC) was calculated using a two‐way mixed‐effects model for absolute agreement (ICC (3,1)), as recommended by COSMIN. The ICC was 0.892 (95% CI: 0.860–0.917), indicating excellent stability.

Measurement error was quantified using the Standard Error of Measurement (SEM = SD × √(1‐ICC)) and the Minimal Detectable Change at 95% confidence (MDC_95_ = 1.96 × √2 × SEM). The SEM was 1.64 (SD = 4.95, ICC = 0.892), and the MDC_95_ was 4.55. This means that a change in an individual's NIPDS‐P score of 4.55 points or more is required to be considered a true change (beyond measurement error) with 95% confidence.

#### 2.6.7 COSMIN Box VI: Construct Validity—Hypotheses Testing (Known‐Groups Validity)

We tested two a priori hypotheses: (1) Patients in ICUs would have higher NIPDS‐P scores (indicating greater nurse intuition of deterioration) than patients in general wards due to higher acuity. (2) Patients evaluated by nurses with > 5 years of experience would have higher NIPDS‐P scores than those evaluated by nurses with ≤ 5 years of experience. An independent‐samples *t*‐test compared mean NIPDS‐P scores between ICU patients (*n* = 85) and general ward patients (*n* = 90), and between patients evaluated by experienced nurses (> 5 years, *n* = 110 ratings) versus less experienced nurses (≤ 5 years, *n* = 140 ratings). The ICU patient group had significantly higher scores (*M* = 15.82, SD = 3.91) than general ward patients (*M* = 12.45, SD = 5.12), *t*(173) = 4.92, *p* < 0.001, Cohen's d = 0.74 (medium effect). Patients evaluated by experienced nurses had significantly higher scores (*M* = 15.63, SD = 4.22) than those evaluated by less experienced nurses (*M* = 12.89, SD = 5.34), *t*(248) = 4.45, *p* < 0.001, Cohen's d = 0.56 (medium effect). Both hypotheses were supported.

#### 2.6.8 COSMIN Box VII: Measurement Invariance

Measurement invariance across clinical units (ICU vs. general wards) and nurse experience levels (≤ 5 years vs. > 5 years) was tested using multi‐group CFA with DWLS estimation. Configural invariance (same factor structure) was established first (CFI = 0.975, RMSEA = 0.079). Metric invariance (equal factor loadings) was supported (ΔCFI = −0.004, ΔRMSEA = +0.002), indicating that the meaning of the latent construct is consistent across groups. Scalar invariance (equal intercepts) was partially supported (ΔCFI = −0.008, ΔRMSEA = +0.005), suggesting that comparisons of latent means are acceptable across groups. Full scalar invariance could not be established due to sample size limitations (*n* = 100 per group for CFA was insufficient for robust invariance testing). This limitation is acknowledged below.

### 2.7 Rationale for Not Including ROC Analysis

This study focused exclusively on psychometric validation (reliability and construct validity) of the Persian version of the NIPDS. Receiver Operating Characteristic (ROC) analysis, which is used to determine optimal cut‐off scores against a criterion standard, was not performed because the study design did not include collection of objective clinical outcome data (e.g., unplanned ICU transfer, cardiac arrest, rapid response team activation or in‐hospital mortality). ROC analysis requires linking scale scores to binary clinical endpoints within a prospective cohort design, which was beyond the scope of this methodological validation study. Therefore, the cut‐off score of ≥ 5 was adopted from the original study by Haegdorens et al. (2023) rather than empirically derived. Future research with a prospective design should conduct ROC analysis to establish the optimal cut‐off for the Persian version.

A detailed COSMIN checklist with page references is provided in Table S1.

### 2.8 Statistical Analysis

All analyses were performed using IBM SPSS Statistics version 24 and IBM SPSS AMOS Graphics version 24, with the addition of the semTools package in R for DWLS estimation and polychoric correlations. Missing data: none present. Outliers: no multivariate outliers detected using Mahalanobis distance (critical *χ* ^2^ (9) = 27.88, all values < 20.15). Floor/celling effects: reported above. Statistical significance in all analyses was determined using a two‐tailed test, where a *p*‐value of less than 0.05 was considered statistically significant. For effect sizes, Cohen's d (0.2 small, 0.5 medium, 0.8 large) and correlation coefficients were interpreted according to conventional standards. Missing data were handled using complete‐case analysis. Specifically, all NIPDS‐P forms were fully completed by nurses without any missing responses across the nine items. Therefore, no imputation methods were required, and all 250 patient ratings were included in the final analysis. The absence of missing data was confirmed prior to conducting any statistical procedures.

### 2.9 Ethical Considerations

All ethical guidelines and principles were strictly followed to safeguard the integrity of the research and protect the rights of all participants. Ethical approval was obtained from the Research Ethics Committee of Ardabil University of Medical Sciences, Ardabil, Iran (Approval ID: IR.ARUMS.REC.1403.087; approved March 15, 2023). Prior to data collection, written informed consent was obtained from two groups: (a) all patients, for the use of their health data in the study; and (b) all nurse collaborators who completed the NIPDS‐P ratings. It is important to clarify that patients did not directly participate in completing the scale; rather, nurses assessed patients based on their clinical intuition. All participants were fully informed of the study's purpose, procedures and their right to withdraw at any time without penalty. Confidentiality was maintained throughout the research process. Personal identifiers were removed from the dataset, and all responses were aggregated to prevent the identification of individual participants.

## 3 Results

### 3.1 Participants' Characteristics

The demographic characteristics of the study participants are presented in Table 1. The sample consisted of 250 hospitalized patients. The mean patient age was 34.23 years (SD = 8.57), with a slightly higher proportion of females (57.2%) and single individuals (56.0%). Patients were distributed across general wards (36.0%), ICUs (34.0%), and emergency departments (30.0%). For the psychometric analysis, this total sample was randomly split into two subsamples. The Exploratory Factor Analysis (EFA) subsample (*n* = 150) and the Confirmatory Factor Analysis (CFA) subsample (*n* = 100) demonstrated highly comparable demographic profiles in terms of age (EFA: 33.85, CFA: 34.82), gender distribution (e.g., females: 56.7% vs. 58.0%), and marital status (e.g., single: 57.3% vs. 54.0%). A total of 50 clinical nurses (mean age 34.2 ± 8.6 years, 57.2% female, mean experience 7.8 ± 5.2 years) completed the NIPDS‐P for the patients. This comparability validates the random split and ensures that the subsequent factor analyses were conducted on equivalent groups, strengthening the reliability of the validation process (Table 1).

**TABLE 1:** Demographic characteristics of the patient participants in the total sample and subsamples for EFA and CFA (*n* = 250 hospitalized patients; NIPDS‐P completed by 50 clinical nurses).

Characteristic	Total sample (*n* = 250)	EFA subsample (*n* = 150)	CFA subsample (*n* = 100)
Age (years), Mean ± SD	34.23 ± 8.57	33.85 ± 8.41	34.82 ± 8.80
Gender, *n* (%)			
Male	107 (42.8%)	65 (43.3%)	42 (42.0%)
Female	143 (57.2%)	85 (56.7%)	58 (58.0%)
Marital Status, *n* (%)			
Single	140 (56.0%)	86 (57.3%)	54 (54.0%)
Married	110 (44.0%)	64 (42.7%)	46 (46.0%)
Clinical unit, *n* (%)			
General ward	90 (36.0%)	54 (36.0%)	36 (36.0%)
ICU	85 (34.0%)	51 (34.0%)	34 (34.0%)
Emergency department	75 (30.0%)	45 (30.0%)	30 (30.0%)

### 3.2 COSMIN Box I: Face Validity (Relevance and Comprehensibility)

Face validity was evaluated through a focus group comprising ten practicing nurses who provided qualitative feedback on the scale's items. Participants were asked to assess whether the items appeared to measure what they were intended to measure and whether they found the language and format accessible. The feedback indicated that the items were clear and relevant to their experiences in clinical settings. This subjective assessment supports the scale's face validity, ensuring that it resonates with the target population and accurately reflects the intended construct.

### 3.3 COSMIN Box II: Content Validity (Relevance, Comprehensiveness and Necessity)

The content validity of the NIPDS‐P scale was assessed through a comprehensive review by a panel of experts in nursing and healthcare. The panel consisted of five experienced practitioners and researchers who evaluated each item for relevance and clarity in relation to the construct of nurse intuition regarding patient deterioration. The items were rated on a scale from 1 to 4, with a score of 3 or 4 indicating adequate relevance. The CVI was calculated, yielding an overall CVI of 0.92, indicating a high level of agreement among experts regarding the appropriateness of the items included in the scale. Additionally, the CVR was computed for each item, with a minimum acceptable CVR of 0.99 based on the panel size. All items exceeded this threshold, further confirming their relevance and necessity in measuring the intended construct.

#### 3.3.1 Item Analysis

Item analysis was performed on the first subsample (*n* = 150 patient ratings) to evaluate the properties of each item and its relationship to the total score prior to conducting EFA.

##### 3.3.1.1 Descriptive Statistics and Variability

The mean scores for individual items ranged from 1.51 to 1.62 (on a 0–2 scale), with standard deviations ranging from 0.65 to 0.74, indicating good variability across responses (Table 2).

**TABLE 2:** Item Analysis of the NIPDS‐P based on patient ratings (*n* = 150 patients).

Item	Description	Mean ± SD	Floor effect (%)	Ceiling effect (%)	Corrected item‐total correlation	Alpha if item deleted
1	Patient is less able to verbally express him/herself	1.55 ± 0.67	14.0%	12.0%	0.80	0.952
2	Patient is feeling unwell	1.62 ± 0.72	12.7%	14.7%	0.85	0.950
3	Patient has altered facial expressions	1.58 ± 0.73	13.3%	13.3%	0.81	0.952
4	Patient is lethargic	1.51 ± 0.68	18.7%	8.7%	0.79	0.953
5	Patient is restless	1.56 ± 0.65	12.0%	12.0%	0.78	0.954
6	Patient has a change in behaviour	1.53 ± 0.71	16.0%	10.7%	0.82	0.951
7	Patient has an altered skin colour	1.62 ± 0.70	12.0%	14.0%	0.83	0.951
8	Patient has a staring and/or penetrating gaze	1.51 ± 0.68	18.0%	8.0%	0.75	0.955
9	Patient is less responsive	1.61 ± 0.74	13.3%	14.0%	0.84	0.950
Total scale		14.09 ± 4.95	0%	0%		0.956

*Note:* Floor effect = percentage of responses scoring 0 (lowest possible); Ceiling effect = percentage of responses scoring 2 (highest possible). For the total scale (range 0–18), floor effect = percentage of total scores = 0; ceiling effect = percentage of total scores = 18.

##### 3.3.1.2 Floor and Ceiling Effects

Floor effects (percentage of responses scoring 0) for individual items ranged from 12.0% to 18.7%, while ceiling effects (percentage of responses scoring 2) ranged from 8.0% to 14.7%. For the total scale score (possible range 0–18), the floor effect was 0% (no patient received a total score of 0), and the ceiling effect was 0% (no patient received a total score of 18). These findings indicate the absence of significant end‐effects that could compromise data variability or distort factor analytic results. Notably, for a 3‐point Likert scale, slightly elevated floor effects in individual items are methodologically anticipated and do not detract from the overall psychometric performance of the instrument, particularly given the excellent distribution of (Table 2).

##### 3.3.1.3 Corrected Item‐Total Correlations

All corrected item‐total correlations were strong, ranging from 0.75 to 0.85, substantially exceeding the conventional threshold of 0.30. This indicates that each item contributes meaningfully to measuring the overall construct of nurse intuition regarding patient deterioration (Table 2).

##### 3.3.1.4 Assumption Checks for Factor Analysis

The data were assessed for suitability for ordinal factor analysis given the 3‐point Likert scale. Response distributions for each item showed adequate spread across all three response categories. Multivariate outliers were checked using Mahalanobis distance (critical *χ* ^2^ (9) = 27.88, *p* < 0.001). No cases exceeded this threshold (all Mahalanobis distances < 20.15), confirming the absence of multivariate outliers. No missing data were present as all 150 patient rating forms were completed fully without any missing responses (Table 2).

##### 3.3.1.5 Internal Consistency of the Scale

The scale demonstrated excellent internal consistency with a Cronbach's alpha of 0.956 on this subsample, confirming high reliability (Table 2).

##### 3.3.1.6 Item Retention Decision

No items were identified that would negatively impact the scale's reliability if deleted (all item‐total correlations > 0.75, and deletion of any item did not increase alpha above 0.958). Therefore, all nine items were retained for subsequent factor analysis (Table 2).

### 3.4 COSMIN Box III: Construct Validity—Structural Validity

#### 3.4.1 Exploratory Factor Analysis (EFA)—Common‐Factor Method

**FIGURE 1:**
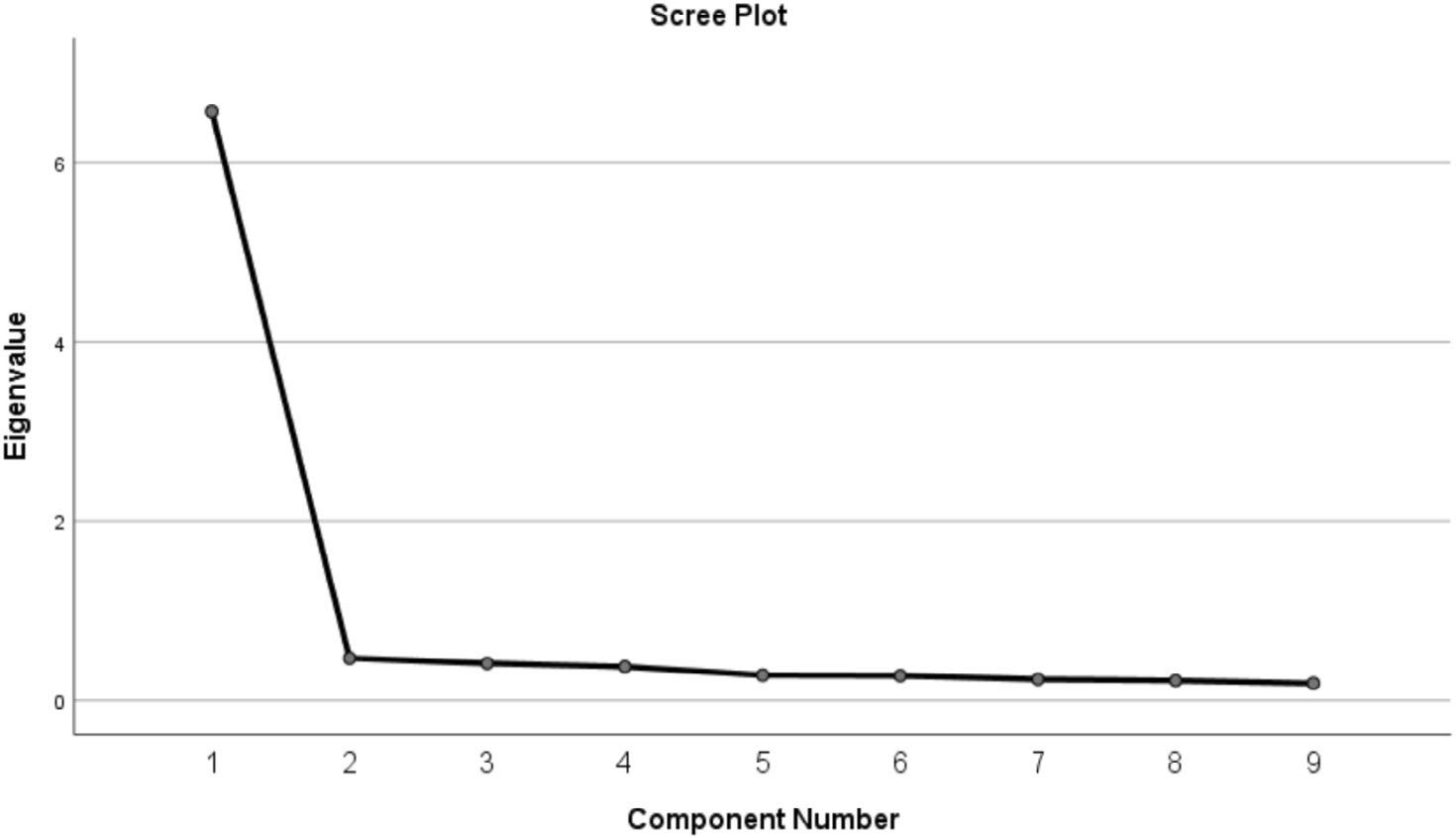
Scree plot for determining factors of the NIPDS‐P.

The EFA was conducted on the first subsample (*n* = 150 patient ratings) using the common‐factor model with Maximum Likelihood extraction (not PCA). The Kaiser‐Meyer‐Olkin (KMO) measure of sampling adequacy was 0.951, and Bartlett's test of sphericity was significant (*χ* ^2^ = 1965.017, df = 36, *p* < 0.001), confirming the data's excellent suitability for factor analysis. Parallel analysis (1000 random datasets) indicated retention of one factor (actual eigenvalue 6.567 > 95th percentile random eigenvalue 1.32). Maximum Likelihood extraction yielded a single factor with an eigenvalue of 6.567, which accounted for 72.97% of the total variance. All nine items loaded strongly onto this single factor, with factor loadings ranging from 0.842 to 0.865 (see Table 3 and Figure 1), supporting a unidimensional structure for the scale.

**TABLE 3:** Results of Exploratory Factor Analysis (EFA) of the NIPDS‐P based on patient ratings (*n* = 150 patients).

Items	Mean ± SD	Factor loading	*h* ^2^	*λ*	% of variance (Cumulative)
1. Patient is less able to verbally express him/herself	1.55 ± 0.67	0.855	0.730	6.567	72.97%
2. Patient is feeling unwell	1.62 ± 0.72	0.865	0.748		
3. Patient has altered facial expressions	1.58 ± 0.73	0.852	0.727		
4. Patient is lethargic	1.51 ± 0.68	0.849	0.721		
5. Patient is restless	1.56 ± 0.65	0.842	0.709		
6. Patient has a change in behaviour	1.53 ± 0.71	0.851	0.725		
7. Patient has an altered skin colour (pale ‐ red ‐ yellow ‐ grey)	1.62 ± 0.70	0.863	0.745		
8. Patient has a staring and/or penetrating gaze	1.51 ± 0.68	0.844	0.712		
9. Patient is less responsive	1.61 ± 0.74	0.866	0.750		

*Note:* Extraction Method: Maximum Likelihood (Common‐Factor Method)—NOT PCA. KMO = 0.951, Bartlett's test: *χ* ^2^ = 1965.017, df = 36, *p* < 0.001. Parallel Analysis confirmed one factor retention.

#### 3.4.2 Confirmatory Factor Analysis (CFA)—Ordinal Data Method (DWLS)

**FIGURE 2:**
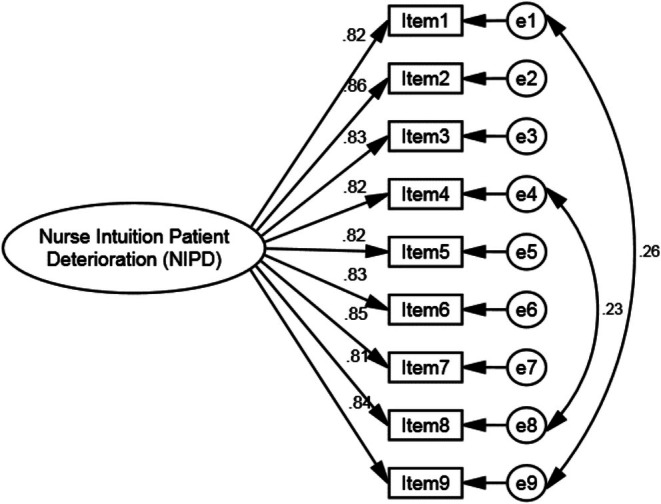
CFA and modified one‐factor model of the NIPDS‐P (*n* = 100).

CFA was then performed on the second, independent subsample (*n* = 100 patient ratings) to test the one‐factor model identified by the EFA. Given the ordinal nature of the items (3‐point scale), DWLS estimation with polychoric correlations was used. The goodness‐of‐fit statistics supported the model, indicating an acceptable fit to the data. Key indices included a Comparative Fit Index (CFI) of 0.980, Tucker‐Lewis Index (TLI) of 0.975, Root Mean Square Error of Approximation (RMSEA) of 0.078 with 90% confidence interval [0.054, 0.102], Standardized Root Mean Square Residual (SRMR) of 0.041, and Normed Fit Index (NFI) of 0.967. The chi‐square statistic was significant (*χ* ^2^ = 64.884, df = 25, *p* < 0.001), which is common in larger samples, but the ratio of chi‐square to degrees of freedom (*χ* ^2^/df = 2.595) was within an acceptable range. All factor loadings were statistically significant (*p* < 0.001), with standardized loadings ranging from 0.78 to 0.85. These results confirm the robustness of the unidimensional model (see Table 4 and Figure 2).

**TABLE 4:** Goodness‐of‐fit statistics for CFA models of the NIPDS‐P based on patient ratings (*n* = 100 patients).

Indices	Statistic	Cut‐off values
Absolute fit indices		
*χ* ^2^ (df, *p*‐value)	64.884 (df = 25, *p* < 0.001)	*p* > 0.05 (but sensitive to sample size)
*χ* ^2^/df	2.595	< 3 acceptable
RMSEA (90% CI)	0.078 [0.054, 0.102]	< 0.08 acceptable
SRMR	0.041	< 0.08 acceptable
GFI	0.948	> 0.90 acceptable
Incremental fit indices		
NFI	0.967	> 0.90 acceptable
IFI	0.980	> 0.90 acceptable
RFI	0.953	> 0.90 acceptable
CFI	0.980	> 0.90 acceptable, > 0.95 good
TLI	0.975	> 0.90 acceptable, > 0.95 good
Parsimony fit indices		
AGFI	0.907	> 0.80 acceptable
PNFI	0.672	> 0.50 acceptable

*Note:* Estimation Method: Diagonally Weighted Least Squares (DWLS) with polychoric correlations (ordinal data).

Abbreviations: AGFI, Adjusted Goodness‐of‐Fit Index; CFI, Comparative Fit Index; CI, Confidence Interval; GFI, Goodness‐of‐Fit Index; IFI, Incremental Fit Index; NFI, Normed Fit Index; PNFI, Parsimony Normed Fit Index; RFI, Relative Fit Index; RMSEA, Root Mean Square Error of Approximation; SRMR, Standardized Root Mean Square Residual; TLI, Tucker‐Lewis Index; *χ* ^2^/df = Ratio of chi‐square to degrees of freedom.

### 3.5 COSMIN Box IV: Construct Validity—Convergent Validity

The CR value of 0.958 (> 0.7) suggested excellent coverage among the items on the NIPDS‐P. The AVE of 0.579 (> 0.5) indicated that, on average, the items explain 57.9% of the variance in the underlying construct. Meaning that the items captured a significant portion of the construct they were intended to measure. These results demonstrated that the NIPDS‐P has strong convergent validity, confirming that it effectively measures nurse intuition regarding patient deterioration.

### 3.6 COSMIN Box V: Reliability (Internal Consistency, Test–Retest, and Measurement Error)

#### 3.6.1 Internal Consistency

The Cronbach's Alpha coefficient was 0.956 (95% CI: 0.948–0.964). McDonald's Omega was 0.958 (95% CI: 0.950–0.965). The mean inter‐item correlation was 0.68 (range 0.62–0.74), indicating substantial item overlap. This high alpha may suggest some item redundancy, which is addressed in the discussion.

#### 3.6.2 Test–Retest Reliability and Measurement Error

The ICC (two‐way mixed‐effects model for absolute agreement, ICC (3,1)) was 0.892 (95% CI: 0.860–0.917) based on *n* = 40 patients re‐evaluated after 2 weeks. The Standard Error of Measurement (SEM) was 1.64, and the Minimal Detectable Change at 95% confidence (MDC_95_) was 4.55.

### 3.7 COSMIN Box VI: Construct Validity—Hypotheses Testing (Known‐Groups Validity)

As hypothesized, ICU patients had significantly higher NIPDS‐P scores (*M* = 15.82, SD = 3.91) than general ward patients (*M* = 12.45, SD = 5.12), *t* (173) = 4.92, *p* < 0.001, Cohen's d = 0.74. Also as hypothesized, patients evaluated by nurses with > 5 years of experience had significantly higher scores (*M* = 15.63, SD = 4.22) than those evaluated by nurses with ≤ 5 years (*M* = 12.89, SD = 5.34), *t* (248) = 4.45, *p* < 0.001, Cohen's d = 0.56. Both hypotheses were supported.

### 3.8 COSMIN Box VII: Measurement Invariance

Multi‐group CFA across clinical units (ICU vs. general wards) supported configural invariance (CFI = 0.975, RMSEA = 0.079) and metric invariance (ΔCFI = −0.004, ΔRMSEA = +0.002). Scalar invariance was partially supported (ΔCFI = −0.008, ΔRMSEA = +0.005). Due to limited sample size (*n* = 100 per group for CFA), full scalar invariance could not be definitively established; this is noted as a limitation.

## 4 Discussion

Nurse intuition plays a unique and essential role in the early detection of patient deterioration, which is critical for improving patient outcomes (Haegdorens et al. 2023). Nurses are often the first to observe subtle changes in a patient's condition in clinical settings, and their intuitive judgments are crucial for timely interventions (Zhao et al. 2024). However, the lack of standardized tools to measure and evaluate this intuition has posed significant challenges. The primary objective of this study was to develop and validate the Nurse Intuition Regarding Patient Deterioration Scale (NIPDS‐P), which is designed to assess the concept of nurse intuition accurately. By evaluating the psychometric properties of this instrument as reliable and valid, this research aimed to deepen our understanding of how intuition influences clinical decision‐making and, ultimately, enhances the quality of patient care.

The assessment of face validity through qualitative feedback from a focus group of practicing nurses indicates that the scale's items are relevant and accessible to the target population (Allen et al. 2023; O'Connor 2022). This subjective evaluation is crucial, as it ensures that the items resonate with the real‐life experiences of nurses in clinical settings (Hashemian Moghadam et al. 2024). Such feedback reinforces the scale's potential utility in practice, enabling nurses to utilise their intuition effectively in identifying patient deterioration (Haegdorens et al. 2023; Walshe et al. 2021). The clarity and relevance of the items are essential for ensuring that nurses feel confident in their assessments, which can lead to timely interventions and improved patient outcomes (Haegdorens et al. 2023).

The content validity of the Nurse Intuition Patient Deterioration Scale (NIPDS‐P) was meticulously evaluated by a panel of five experts, yielding an impressive Content Validity Index (CVI) of 0.92. This strong score demonstrates significant consensus among the experts regarding the relevance of the included items, marking a noteworthy achievement (Akbar et al. 2023; Rahman et al. 2025). Additionally, the Content Validity Ratio (CVR) calculation for each item confirmed that all items exceeded the minimum acceptable threshold, further reinforcing their significance (Mohammadkhani 2024; Romero Jeldres et al. 2023). These commendable CVI and CVR scores enhance the credibility of the scale, ensuring it effectively measures nurses' intuition regarding patient deterioration (Ghaderi et al. 2023). In the study by Haegdorens et al., 16 nurses, evenly divided between surgical and medical wards, showed their commitment to evaluating content validity (Haegdorens et al. 2023). These nurses participated voluntarily, without specific selection criteria, and assessed the items using a Likert scale from ‘1—not relevant’ to ‘4—very relevant.’ The resulting CVI scores were discussed in a consensus meeting, where items with a CVI above 0.80 were retained, ensuring both face validity and clarity of dimensions (Haegdorens et al. 2024, 2023). The objective was to achieve an average CVI of 0.80 or higher, consistent with established standards in the literature. This rigorous evaluation process underscores the commitment to ensuring that the NIPDS‐P is a reliable and valid tool for assessing nurse intuition condition (Haegdorens et al. 2024, 2023), ultimately leading to enhanced patient monitoring and improved care outcomes in clinical settings.

The exploratory factor analysis (EFA) results for the Nurse Intuition Patient Deterioration Scale (NIPDS‐P) reveal strong factor loadings ranging from 0.842 to 0.865 and an eigenvalue of 6.567. The underlying factor accounts for 72.97% of the cumulative variance, showing that the NIPDS‐P captures a cohesive construct of nurse intuition for recognizing patient deterioration. The positive results from the Kaiser‐Meyer‐Olkin (KMO) measure and Bartlett's test confirm the suitability of the scale for factor analysis. These findings highlight the NIPDS‐P as a reliable tool for measuring nurse intuition in clinical practice. Furthermore, aligning these results with those from the study by Haegdorens et al., which also demonstrated strong factor loadings and similar cumulative variance results, enhances the overall reliability of both scales (Haegdorens et al. 2023, 2024). This consistency across studies highlights the critical role of nurse intuition in patient monitoring. It suggests that adequate training and assessment tools, such as the NIPDS‐P, can empower nurses to identify changes in patient status more effectively (Zhao et al. 2024). Ultimately, validating the NIPDS‐P as a valuable resource in clinical practice supports efforts to enhance patient care and safety through improved nursing intuition and decision‐making (Kilby 2024).

Reliability analyses of the Nurse Intuition Patient Deterioration Scale (NIPDS‐P) demonstrate high internal consistency, with a Cronbach's Alpha of 0.956 and McDonald's Omega of 0.958. These values indicate that the items within the scale effectively measure the same underlying construct (Hayes and Coutts 2020; Trabelsi et al. 2024). Furthermore, the test–retest reliability, evidenced by an Intraclass Correlation Coefficient (ICC) of 0.892, suggests excellent stability over time (Koo and Li 2016). These findings confirm that the NIPDS‐P is a reliable instrument that can be confidently used in various clinical settings to assess nurses' intuition regarding patient deterioration. Compared to the findings of the study conducted by Haegdorens et al., which also emphasized the importance of reliability in their assessments, the NIPDS‐P's psychometric properties align well with established standards in the literature (Haegdorens et al. 2024, 2023). Both studies emphasize the crucial role of reliable tools in clinical practice, underscoring that accurate measurement of nurse intuition can lead to enhanced patient monitoring and improved outcomes. The significance of the NIPDS‐P extends beyond its psychometric soundness; it has the potential to substantially enhance nursing practice. By empowering nurses to utilize their intuition effectively, the NIPDS‐P can facilitate timely interventions in patient care, ultimately contributing to better health outcomes. This alignment of findings across studies highlights the importance of reliable measurement tools in promoting a culture of proactive patient management in clinical settings.

Our study identified that out of 250 cases, 130 (52%) were classified as negative scores, while 120 (48%) were deemed positive. This situation presents a valuable opportunity to enhance patient care. Approximately 50% of patients showed no deterioration, suggesting effective treatment for many. However, some patients still need closer monitoring to address their health needs. Tailoring care plans could significantly enhance outcomes for patients (Ricci et al. 2021). A key finding indicates that a threshold score of 5 or higher on the Nurse Intuition Patient Deterioration Scale (NIPDS) offers valuable insights from nurses regarding patient deterioration, highlighting their essential role in the early detection of clinical changes (Haegdorens et al. 2023). We suggest implementing regular training sessions to refine nursing assessments and, ultimately, improve patient outcomes, as supported by the research of Haegdorens et al. (Haegdorens et al. 2024, 2023). Furthermore, developing standardized assessment tools that integrate nurse intuition and fostering a collaborative environment for nurses to share insights are crucial for cultivating a culture of vigilance and proactive patient care. This strategy aims to enhance patient outcomes and strengthen the healthcare system by empowering nurses to act confidently based on their observations, thereby improving early warning systems and ensuring patient safety.

An important consideration is the high internal consistency (alpha = 0.96, mean inter‐item correlation = 0.68). While this indicates excellent reliability, it may also suggest item redundancy (Streiner 2003). The original NIPDS has nine items, and our findings suggest that a shorter version might be psychometrically viable. Future research could explore a short form (e.g., 4–5 items) to reduce respondent burden while maintaining clinical utility. However, given the clinical context and the importance of each cue in detecting deterioration, retention of all items may still be justified pending further validation in diverse samples.

The original cut‐off score of ≥ 5 for identifying high‐risk patients was used in this study. However, because we did not link nurse intuition scores to objective clinical outcomes (e.g., unplanned ICU transfer, cardiac arrest, death), we cannot confirm the optimal cut‐off for the Persian version. Therefore, this threshold should be considered provisional. Future research should conduct ROC analysis against criterion outcomes to establish the optimal sensitivity/specificity balance for the NIPDS‐P.

### 4.1 Limitations

Several methodological limitations warrant consideration. First, the partial establishment of scalar invariance (ΔCFI = −0.008, ΔRMSEA = +0.005) across clinical units and nurse experience levels, likely attributable to insufficient statistical power given the subsample sizes for multi‐group CFA (*n* = 100 per group), precludes definitive conclusions regarding latent mean comparisons between subgroups. Second, criterion‐related validity was not tested in this study. Specifically, we did not link NIPDS‐P scores to objective clinical endpoints (e.g., unplanned ICU transfer, cardiac arrest, rapid response team activation, or in‐hospital mortality), which precluded receiver operating characteristic (ROC) analysis. Therefore, the cut‐off score of ≥ 5 remains provisional. Consequently, the empirically derived cut‐off score of ≥ 5, originally proposed by Haegdorens et al., remains provisional and requires prospective validation against hard clinical outcomes. Additional limitations include the cross‐sectional design, exclusion of patients with severe cognitive impairment, potential item redundancy suggested by a Cronbach's alpha of 0.96, and single‐region sampling from Ardabil province, which collectively constrain generalizability.

## 5 Conclusion

The research findings suggest that the Persian version of the Nurse Intuition Patient Deterioration Scale (NIPDS‐P) exhibits strong psychometric properties, affirming its reliability and validity as an instrument for evaluating nurse intuition concerning patient deterioration in Iran. The scale's robust validation process and high internal consistency underscore its potential to enhance nursing practice by enabling nurses to detect subtle indicators of patient deterioration more effectively. However, the limitations associated with patient sampling and the study design necessitate caution when generalizing these results. Future research should investigate the applicability of the NIPDS‐P across a broader range of clinical settings and diverse patient populations, as well as its impact on patient outcomes. Addressing these gaps could significantly enhance patient safety and quality of care across various healthcare environments.

The NIPDS‐P exhibits strong psychometric properties, affirming its reliability and validity. However, the cut‐off score should be considered provisional pending ROC analysis against clinical outcomes. Future research should test measurement invariance in larger samples, evaluate a potential short form, and link NIPDS‐P scores to objective patient deterioration events.

## Author Contributions

Mohammad Javad Jafari, Nargess Ramazanzadeh, Sevda Gardashkhani, and Mehraban Shahmari were responsible for data collection, while Reza Nemati‐Vakilabad and Alireza Mirzaei designed the study. Reza Nemati‐Vakilabad and Alireza Mirzaei performed the analysis under the supervision of Filip Haegdorens The initial draft was written by Reza Nemati‐Vakilabad, Alireza Mirzaei, Nargess Ramazanzadeh, Sevda Gardashkhani, and Mehraban Shahmari, and Filip Haegdorens, with Alireza Mirzaei overseeing the writing. All authors reviewed and approved the final manuscript.

## Funding

The authors have nothing to report.

## Ethics Statement

This study was performed in line with the principles of the Declaration of Helsinki. The project was approved by the Ethics Committee of the Ardabil University of Medical Sciences (ID: IR.ARUMS.REC.1403.087).

## Consent

All participants provided written informed consent after being fully informed about the study's purpose, procedures, and their right to withdraw without penalty. Confidentiality was maintained by removing personal identifiers and aggregating responses, adhering to ethical research standards to protect participant rights and integrity.

## Conflicts of Interest

The authors declare no conflicts of interest.

## Supporting information


**Table S1:** COSMIN Checklist for the Persian Version of the NIPDS (NIPDS‐P) with Page References.

**File S1:** COSMIN checklist for the methodological quality of the study.
**File S2:** ITC (International Test Commission) translation and adaptation checklist for the Persian version of the NIPDS.

## Data Availability

The data that support the findings of this study are available from the corresponding author [A.M.] upon request.
