# Supplementary material for: Validation and Psychometric Evaluation of the Persian Version of the Nurse Intuition Patient Deterioration Scale: A Methodological Study
Source: Nurs Open. 2026 May 25;13(5):e70616. doi: 10.1002/nop2.70616 (PMC13240193; doi:10.1002/nop2.70616)
Supplement: Supplementary file 1 — Table S1: COSMIN Checklist for the Persian Version of the NIPDS (NIPDS‐P) with Page References. [file NOP2-13-e70616-s002.docx]

**Supplementary Table 1. COSMIN Checklist for the Persian Version of the NIPDS (NIPDS-P) with Page References**

| **Domain** | **Measurement Property** | **Aspect of measurement property** | **Response (Yes/No/N/A)** | **Page Reference** | **Location in Manuscript** |
| --- | --- | --- | --- | --- | --- |
| **Reliability** | Internal consistency | — | **Yes ✓** | **pp. 12-13** | Results: Internal consistency |
|  | Reliability (test-retest) | — | **Yes ✓** | **p. 12** | Results: Test-retest reliability |
|  | Measurement error | — | **Yes ✓** | **p. 12** | Results: Measurement error (SEM, MDC) |
| **Validity** | Content validity | — | **Yes ✓** | **p. 9** | Methods: COSMIN Box II |
|  |  | Face validity | **Yes ✓** | **p. 8** | Methods: COSMIN Box I |
|  | Construct validity | Structural validity | **Yes ✓** | **pp. 10-12** | Results: EFA & CFA |
|  |  | Hypotheses testing | **Yes ✓** | **p. 13** | Results: Hypotheses testing (known-groups) |
|  |  | Cross-cultural validity | **Yes ✓** | **pp. 7-8** | Methods: Translation and cross-cultural adaptation |
|  | Criterion validity | — | **No ✓** | **p. 15** | Limitations (stated as limitation) |
| **Responsiveness** | Responsiveness | — | **N/A ✓** | **p. 15** | Not applicable (cross-sectional design) |
| **Interpretability** | Interpretability | — | **Yes ✓** | **pp. 10, 13-14** | Results: Mean scores, floor/celling effects, cut-off discussion |

**Note:** This checklist follows COSMIN guidelines for methodological studies of measurement properties. Page references correspond to the main manuscript.
