# Supplementary material for: Validation and Psychometric Evaluation of the Persian Version of the Nurse Intuition Patient Deterioration Scale: A Methodological Study
Source: Nurs Open. 2026 May 25;13(5):e70616. doi: 10.1002/nop2.70616 (PMC13240193; doi:10.1002/nop2.70616)
Supplement: Supplementary file 2 — File S1: COSMIN checklist for the methodological quality of the study. File S2: ITC (International Test Commission) translation and adaptation checklist for the Persian version of the NIPDS. [file NOP2-13-e70616-s001.docx]

**ITC Translation/Adaptation Checklist – Completed Form**

**Study Title:** Validation and Psychometric Evaluation of the Persian Version of the Nurse Intuition Patient Deterioration Scale (NIPDS-P)

**Reference:** Hernández, A., Hidalgo, M. D., Hambleton, R. K. & Gómez-Benito, J. (2020). International Test Commission guidelines for test adaptation: A criterion checklist. *Psicothema*, 32(3), 390-398.

**1. PRE-CONDITION GUIDELINES**

| Criterion | Achievement Level | Justification / Evidence |
| --- | --- | --- |
| **PC2-1 (G2, C1):** Provide theoretical and empirically-based evidence that the construct of interest is relevant to the target population. | **Excellent** | Theoretical reasons (literature on nurse intuition in deterioration, Haegdorens et al., 2023, 2024) and empirical evidence (clinical relevance confirmed by expert panel of 10 Iranian nurses and 5 healthcare experts) are provided. |
| **PC2-2 (G2, C2):** Justify that translation/adaptation is preferable to creating a new test. | **Excellent** | A group of experts (n=5) in nursing, psychometrics, and Iranian culture checked the construct definition, dimensionality, and items. They confirmed complete overlap in the construct and adequacy of source items for the Iranian population. |
| **PC1-1 (G1, C3):** Obtain written permission from copyright owner. | **Excellent** | Written permission was obtained from Professor Haegdorens (original developer) via email correspondence (dated [insert date]). Permission included allowance for adaptation, cultural modifications, and publication of results. |
| **PC3-1 (G3, C4):** Check cultural and linguistic differences before adaptation. | **Excellent** | Cultural and linguistic differences (e.g., familiarity with "staring gaze," "altered facial expressions" in Iranian context) were systematized and documented based on expert opinions (n=5 Iranian experts) and empirical evidence (cognitive interviews with 15 nurses). |

**2. DEVELOPMENT GUIDELINES**

| Criterion | Achievement Level | Justification / Evidence |
| --- | --- | --- |
| **TD1-1 (G4, C5):** Form a multidisciplinary team. | **Acceptable** | Team included: 2 professional translators (bilingual Farsi-English), 2 nursing experts (construct experts), 1 psychometrician (test construction expert), and 1 methodologist. Total 6 members with overlapping expertise. |
| **TD2-1 (G5, C6):** Use recommended translation designs and justify the choice. | **Excellent** | Forward translation (2 independent translators) → Synthesis → Back-translation (2 independent translators) → Expert committee review. This design was chosen to detect discrepancies directly in the target language (ITC, 2017 recommendation). |
| **TD2-2 (G5, C7):** Independent translators and committee to resolve discrepancies. | **Excellent** | Two translators worked on each design phase (forward and back). An independent committee (n=6 experts) reviewed all versions and resolved discrepancies through consensus meetings. |
| **TD3-1 (G6, C8):** Ensure instructions are clear and comprehensible. | **Excellent** | Expert committee reviewed and approved instructions. Pilot study (n=15 nurses) with cognitive interviews confirmed clarity and comprehensibility. |
| **TD3-2 (G6, C9):** Ensure item content is clear and similarly understood. | **Excellent** | Expert committee reviewed and approved item content. Pilot testing (n=15 nurses) with cognitive interviews confirmed that items were clearly understood and comparable to the original. |
| **TD4-1 (G7, C10):** Ensure item format, response options, scoring rubrics are similar. | **Excellent** | Experts agreed (with justification) that the 3-point Likert format (0-2), response options, and scoring rubrics were highly similar to the original version. |
| **TD4-2 (G7, C11):** Ensure target population familiarity with procedures. | **Excellent** | Previous studies in Iran have used similar Likert scales in nursing research. Pilot study results (n=15) showed no difficulties with procedures. |
| **TD5-1 (G8, C12):** Check psychometric quality in pilot sample. | **Excellent** | Item analysis (difficulty, discrimination, corrected item-total correlations: 0.75-0.85), reliability (Cronbach's alpha = 0.956, Omega = 0.958), and validity (EFA, CFA) were conducted. Results were excellent according to standard criteria. |

**3. CONFIRMATION (EMPIRICAL ANALYSIS) GUIDELINES**

| Criterion | Achievement Level | Justification / Evidence |
| --- | --- | --- |
| **C1-1 (G9, C13):** Ensure sample size is adequate for statistical analyses. | **Excellent** | Total sample N=250 (EFA subsample n=150; CFA subsample n=100). Sample size exceeds minimum requirements for EFA (n=150 > 5 cases per item) and CFA (n=100 > recommended minimum of 100 for DWLS estimation). |
| **C1-2 (G9, C14):** Ensure source and target samples are comparable. | **Acceptable** | Source sample (Belgian, Haegdorens et al.) and target sample (Iranian) have similar characteristics (hospitalized patients, nurse raters). Differences in culture and language are the primary variables of interest. |
| **C2-1 (G10, C15):** Use statistical procedures to ensure construct equivalence. | **Excellent** | Both internal structure (EFA: one factor explaining 72.97% variance; CFA: CFI=0.980, TLI=0.975, RMSEA=0.078) and nomological network (hypotheses testing with known groups: ICU vs. general wards, experienced vs. less experienced nurses) support construct equivalence. |
| **C2-2 (G10, C16):** Check for method equivalence. | **Acceptable** | Instrument characteristics (identical format), administration process (standardized), and sample characteristics (comparable) were checked. No significant method effects were identified. |
| **C2-3 (G10, C17):** Assess DIF across groups. | **N/A** | DIF analysis was not conducted as cross-cultural comparison between source and target populations was not the primary objective of this validation study. This is noted as a limitation for future research. |
| **C2-4 (G10, C18):** Analyze reasons for DIF if detected. | **N/A** | Not applicable (no DIF analysis conducted). |
| **C3-1 (G11, C19):** Provide adequate reliability indicators with SEM. | **Excellent** | Internal consistency (Cronbach's alpha = 0.956, Omega = 0.958), test-retest reliability (ICC = 0.892, 95% CI: 0.860-0.917), and Standard Error of Measurement (SEM = 1.64) are reported. |
| **C3-2 (G11, C20):** Provide validity evidence consistent with intended use. | **Excellent** | Content validity (CVI=0.92, CVR>0.99), structural validity (EFA and CFA), and hypotheses testing (known-groups validity) are provided, supporting the intended use of the scale. |
| **C3-3 (G11, C21):** Ensure norms are adequate for target population. | **Acceptable** | The cut-off score (≥5) from the original version is used provisionally pending ROC analysis. Specific norms for the Iranian population were not developed in this study; this is noted as a limitation. |
| **C4-1 (G12, C22):** Use linking designs when DIF is present. | **N/A** | Not applicable (no DIF analysis or score linking conducted). |

**4. ADMINISTRATION GUIDELINES**

| Criterion | Achievement Level | Justification / Evidence |
| --- | --- | --- |
| **A1-1 (G13, C23):** Check administration materials against development guidelines. | **Excellent** | All criteria in TD3 to TD5 were met at "Excellent" level. The original administration protocol was followed. |
| **A2-1 (G14, C24):** Ensure testing conditions are standardized across groups. | **Excellent** | Administration mode (paper-based questionnaire), time restrictions (no time limit, but typical completion time 5-7 minutes), and instructions were standardized for all nurse raters. |
| **A2-2 (G14, C25):** Ensure administrators have required credentials. | **Acceptable** | Nurse raters (n=50) were registered nurses with at least 6 months of clinical experience. Training on the NIPDS-P was provided, and competency was verified before data collection. |

**5. SCORE SCALES AND INTERPRETATION GUIDELINES**

| Criterion | Achievement Level | Justification / Evidence |
| --- | --- | --- |
| **SSI2-1 (G16, C26):** Ensure measurement equivalence before comparing scores. | **Acceptable** | Configural and metric invariance were supported. Scalar invariance was partially supported (ΔCFI = -0.008, ΔRMSEA = +0.005). Full scalar invariance could not be established due to sample size limitations. |
| **SSI1-1 (G15, C27):** Consider cultural/linguistic factors when interpreting differences. | **Excellent** | Cultural and linguistic differences were systematized (PC3-1). The provisional cut-off (≥5) is interpreted with caution, and recommendations for future ROC analysis are provided. |

**6. DOCUMENTATION GUIDELINES**

| Criterion | Achievement Level | Justification / Evidence |
| --- | --- | --- |
| **Doc-1-1 (G17, C28):** Create documentation with all relevant information. | **Excellent** | This ITC checklist, the COSMIN checklist, and the complete manuscript provide comprehensive documentation of: (a) construct relevance, (b) cultural/linguistic differences, (c) bias prevention steps, (d) deviations from original, (e) team members and design, (f) sample characteristics, (g) psychometric properties, (h) equivalence results. |
| **Doc-2-1 (G18, C29):** Ensure test manual and materials are clear. | **Acceptable** | The Persian NIPDS includes clear instructions, description of scoring (0-18, cut-off ≥5 provisional), and examples. A full test manual is planned for future development. |

**Explanation of Each Translation Step (as per ITC Guidelines)**

| Step | Description | ITC Criterion |
| --- | --- | --- |
| **Step 1: Initial Forward Translation** | Two independent bilingual translators (native Farsi speakers, proficient in English) translated the original NIPDS into Farsi. One translator was a nurse (familiar with the construct), the other was a professional translator (naïve to the scale). | TD2-1, TD2-2 |
| **Step 2: Synthesis of Forward Translations** | The two translators and the research team reviewed T1 and T2, resolved discrepancies in terminology and cultural meaning, and produced a single reconciled Farsi version (T-12). | TD2-2 |
| **Step 3: Back-Translation** | Two independent bilingual translators (native English speakers, fluent in Farsi, unaware of the original instrument) back-translated the reconciled Farsi version into English (BT1 and BT2). | TD2-1 |
| **Step 4: Expert Committee Review** | An expert committee (forward/back translators, methodologist, language professional) compared original NIPDS with BT1/BT2 and T-12, resolving semantic, idiomatic, and conceptual inconsistencies to produce a pre-final version. | TD2-2, TD1-1 |
| **Step 5: Review by Original Developer** | The pre-final Persian version was submitted to Professor Haegdorens (original developer), who confirmed that the translated version maintained the original instrument's intent, conceptual integrity, and item structure. | PC1-1 |
| **Step 6: Pretesting and Cognitive Debriefing** | The pre-final version was administered to 15 practicing nurses, who provided feedback on clarity, comprehensibility, and cultural appropriateness. Minor wording adjustments were made to finalize the Persian NIPDS. | TD3-1, TD3-2, TD5-1 |

**Ethical Considerations (IRB Details)**

**Ethical approval** was obtained from the **Research Ethics Committee of Ardabil University of Medical Sciences, Ardabil, Iran** (Approval ID: **IR.ARUMS.REC.1402.045**, approved **15 March 2023**).

**Written informed consent** was secured from all patient participants and nurse collaborators prior to data collection. Participants were fully informed of the study's purpose, procedures, and their right to withdraw without penalty. Confidentiality was maintained throughout; personal identifiers were removed, and all responses were aggregated.
